# Hypergraph reconstruction from uncertain pairwise observations

**DOI:** 10.1038/s41598-023-48081-w

**Published:** 2023-12-04

**Authors:** Ariane Lizotte, Jean-Gabriel Young, Antoine Allard

**Affiliations:** 1 Département de Physique, de génie Physique et d’optique, Université Laval, Québec, G1V 0A6 Canada; 2 Centre Interdisciplinaire en Modélisation Mathématique, Université Laval, Québec, G1V 0A6 Canada; 3 Department of Mathematics and Statistics, University of Vermont, Burlington, VT 05405 USA; 4 Vermont Complex Systems Center, University of Vermont, Burlington, VT 05405 USA

**Keywords:** Complex networks, Applied mathematics

## Abstract

The network reconstruction task aims to estimate a complex system’s structure from various data sources such as time series, snapshots, or interaction counts. Recent work has examined this problem in networks whose relationships involve precisely two entities—the pairwise case. Here, using Bayesian inference, we investigate the general problem of reconstructing a network in which higher-order interactions are also present. We study a minimal example of this problem, focusing on the case of hypergraphs with interactions between pairs and triplets of vertices, measured imperfectly and indirectly. We derive a Metropolis-Hastings-within-Gibbs algorithm for this model to highlight the unique challenges that come with estimating higher-order models. We show that this approach tends to reconstruct empirical and synthetic networks more accurately than an equivalent graph model without higher-order interactions.

## Introduction

Networks are a simple yet powerful model for the intricate structure of complex systems, in which interactions between any pair of the system’s constituting elements can be directly interpreted as edges between the corresponding vertices of a graph. In typical network analyses, these pairwise interactions will initially be unknown as we cannot observe them directly; one must instead define a model of what is and is not an interaction and put this model to the data to identify the relevant network. For instance, we might define a pollinator and a plant species as interacting if a pollinator prefers a particular species over others. This definition will then let us infer a plant-pollinator interaction network by observing how often each pollinator visits each plant and processing the data with an appropriate statistical model^1,2^.

Numerous methods have been proposed to perform this critical step of the network analysis process, commonly called graph reconstruction, network inference and network reverse engineering. They span a broad range of statistical and machine learning techniques and are often tailored to the specific field for which they have been developed^3^. Gene regulatory networks, for instance, have been reconstructed with methods ranging from random forests^4^ and support vector machine algorithms^5^ to methods based on Pearson correlation in temporal windows^6^, hypothesis testing^7^, least angle regression^8^ and ordinary differential equations^9^. Bayesian frameworks based on genomic features^10^ or random-walk-based algorithms^11^ have been used to estimate protein-protein interaction networks; while brain networks have been measured with a vast range of methods like cross-frequency phase synchronization^12^, Granger causality^13^, and matrix-regularized network learning frameworks^14^. More general methods have also been developed to reconstruct diverse datasets^15–19^.

While useful, graphs are fundamentally limited to encoding dyadic connections and higher-order interactions aren’t always reducible to a set of pairwise ties^20,20,21^. For example, empirical evidence shows that accounting for such higher-order interactions can enhance models of cortical dynamics^22^, of biodiversity^23–25^, and of social group formation^26^. If they are to reap the benefits of such representations, network science methods should be able to handle higher-order interactions whenever dyadic relationships are insufficient.

There has been significant recent progress in adapting network science methods to higher-order representations^27^, but the reconstruction of higher-order structures has only been tackled more recently. Prior work construct simplicial complexes from cliques of a given graph^28,29^ or from proximity of vertices in a latent metric space^30,31^, use network data to make inferences about possible higher-order structures^32^, filters on incomplete hyperedge data^33^ or apply expectation maximization on binary time series to retrieve edges and 2-simplices^34^. And although higher-order interactions of a network can be seen as form of overlapping communities^35^, community detection is generally interested in mesoscale or large-scale communities. Hence, no method to date can simultaneously handle reconstruction and uncertainty in the pairwise measurements.

This paper introduces a general Bayesian framework to infer higher-order structural interactions from imperfect pairwise measurements. We illustrate its use with a minimal example of this problem, focusing on the case of hypergraphs with interactions between pairs and triplets of vertices, measured imperfectly and indirectly. Instead of providing a point estimate, this framework offers a distribution of the possible hypergraphs compatible with all the available observations. The range of structures provided by this distribution allows us to compute error bars for various network measurements and the outcomes of network processes^36^. We also present a network model that encodes the projection of hyperedges as different types of pairwise interactions, and use it to analyze the impact correlations induced by higher-order interactions can have on the inference outcome. To this end, we consider a real-world dataset as well as synthetic observations obtained from empirical hypergraphs. Finally, we investigate and discuss the limitations of these two frameworks.

## Methods

Let us assume that we possess some measurements $X=[x_{ij}]_{i,j=1,\ldots ,n}$ of the pairwise interactions of the units of a complex system composed of *n* elements. In general reconstruction problems, these observations could take on many forms, such as time series correlation of brain regions^37^ or the direct observation of the presence (or absence) of edges in a networked system^15^, to name only two examples. To keep our presentation of the methods concrete, we will focus on the case where $x_{ij}$ is an integer number of observed interactions for vertices *i* and *j*. Our objective is to infer the interactions in a hidden latent structure $\mathcal {S}$ under the assumption that these interactions shape the observed behavior of the system (i.e., the measurements). This latent structure could be any type of structural representation such as graphs, simplicial complexes, or hypergraphs.

We expect the observation data to be uncertain, meaning that remeasuring the system could lead to different values *X* for the same underlying structure $\mathcal {S}$. For instance, two pairwise observations $x_{ij}$ and $x_{rs}$ could be identical even if the pair (*i*, *j*) interacts in $\mathcal {S}$ while (*r*, *s*) does not. To account for these fluctuations, we develop a Bayesian inference framework, a fully probabilistic approach producing a probability distribution over the different structures $\mathcal {S}$ compatible with the data *X*.

### Data model

Our framework first requires to specify the likelihood $P(X|\mathcal {S}, \mu )$, which expresses how the observations *X* are related to the latent structure $\mathcal {S}$ and any additional parameters of the observation processes μ. We assume that the structure $\mathcal {S}$ encodes three types of symmetrical interactions: each pair (*i*, *j*) can interact weakly ($\ell _{ij}=1$), interact strongly ($\ell _{ij}=2$) or not interact ($\ell _{ij}=0$). For instance, measurements *X* of a social network could be the number of conversations recorded between acquaintances ($\ell _{ij}=1$), friends ($\ell _{ij}=2$) or strangers ($\ell _{ij}=0$).

**Figure 1:**
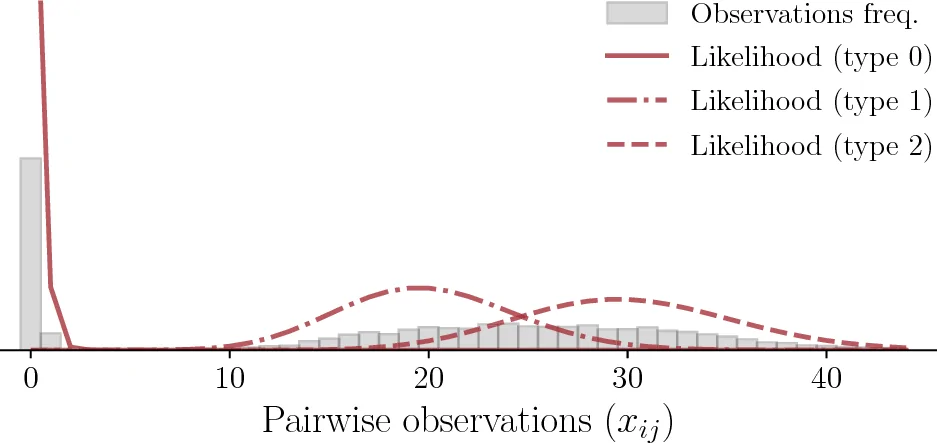
Illustration of a typical distribution of pairwise interactions *X* produced by the data model. The frequencies of the pairwise interactions are shown in gray. The contribution of each type of interaction to the likelihood is shown in red.

Supposing that two distinct measurements $x_{ij}$ and $x_{rs}$ are not correlated, and that every $x_{ij}$ is the outcome of numerous independent observations of an ongoing measurement process with constant success rate $\mu _{\ell _{ij}}$ determined by the interaction type, the likelihood is a product of Poisson distributions

1 $$\begin{aligned} P(X|\mathcal {S}, \mu ) = \prod _{i<j} \frac{\mu _{\ell _{ij}}^{x_{ij}}}{x_{ij}!}e^{-\mu _{\ell _{ij}}}, \end{aligned}$$

where $\mu = (\mu _0, \mu _1, \mu _2)$. Figure 1 illustrates the distribution of pairwise observations modeled by Eq. (1).

Note that we make these assumptions to provide the most simple illustration of our inference framework for pedagogical purposes. In fact, any particular empirical dataset will require its own data model determined through iterative experimentation^38^; the Bayesian inference process for a particular empirical dataset rarely generalizes to other datasets^19^.

### Structural models

The next step is to specify the latent structural model $P(\mathcal {S}|\phi )$, which is a prior probability on each interaction $\ell _{ij}$ conditioned on some additional hyperparameters collectively denoted by ϕ. This distribution encodes our hypothesis on the structure of interactions of the system before we make any measurements. For instance, we might expect person *i* to be more likely to develop a friendship with person *j* than with person *k* because *i* and *j* live in the same neighborhood.

To highlight the role of latent higher-order interactions in the reconstruction procedure (or lack thereof), we consider two models for the structure $\mathcal {S}$: a hypergraph model ($\mathcal {S}=H$) and a categorical-edges model with a graph structure ($\mathcal {S}=G$).

#### Hypergraph model

We define the hypergraph structure H=(V,E,T) as a set of vertices *V* with 2-edges *E* and 3-edges *T*. We limit the size of the hyperedges to 3 for the sake of simplicity, although larger hyperedges could easily be considered by adapting the data model in Eq. (1) accordingly. We opt for a simple hypergraph model in which the existence of each hyperedge is conditionally independent from the others. Denoting as *p* (*q*) the probability of existence of 3-edges (2-edges), the prior probability of *H* is

2 $$\begin{aligned} P(H|\phi _H) = q^{h_1} (1-q)^{\left( {\begin{array}{c}n\\ 2\end{array}}\right) -h_1} p^{h_2} (1-p)^{\left( {\begin{array}{c}n\\ 3\end{array}}\right) -h_2}, \end{aligned}$$

where $\phi _H=\{p,q\}$ are the structure hyperparameters, $h_1=|E|$ is the number of 2-edges and $h_2=|T|$ is the number of 3-edges.

We connect this structure to the data model by assigning a type $\ell _{ij}$ to each pair of vertices as

3 $$\begin{aligned} \ell _{ij}= {\left\{ \begin{array}{ll} 2 &{} \text {if }(i,j) \in \Delta ,\\ 1 &{} \text {if }(i,j) \in E \text { and if } (i,j)\not \in \Delta ,\\ 0 &{} \text {otherwise,} \end{array}\right. } \end{aligned}$$

where Δ is the set of pairs covered by a 3-edge

4 $$\begin{aligned} \Delta = \{ (i, j)\ |\ \exists \ k \text { s.t. } (i,j,k) \in T \}. \end{aligned}$$

To make further progress, we must make a few arbitrary choices since the full model—the joint distribution of the data and latent structure—can be re-parametrized in ways that do not affect the distribution over labels and, therefore, over data. These symmetries will cause identifiability problems when we use the model to make inferences about latent hypergraphs, so we address them immediately.

**Figure 2:**
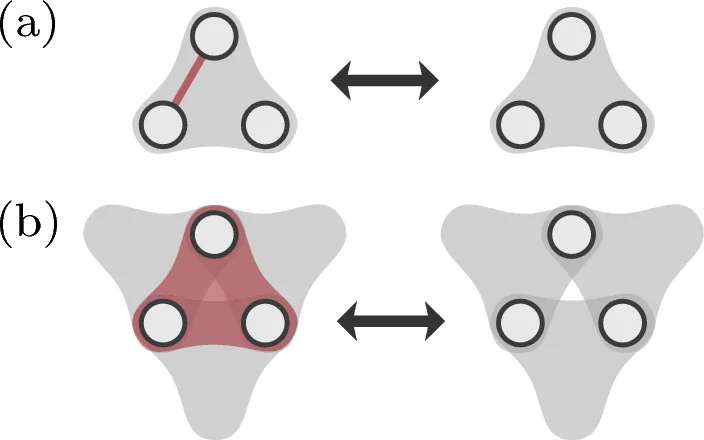
Examples of structural configurations with hidden hyperedgess. The presence or absence of the (**a**) 2-edge and (**b**) 3-edge shown in red does not alter the type of pairwise interaction $\ell _{ij}$ of the vertices, which is the same for all configurations. Hence, the likelihood in Eq. (1) has the same value, and we say that these red hyperedges are *hidden* by the other 3-edges.

First, since the mapping from hypergraph to labels is lossy, the presence of some hyperedges can be *hidden* by others. For example, if vertices *i* and *j* are connected by both a 2-edge and a 3-edge (see Fig. 2a), then the pairwise interaction will be considered of type $\ell _{ij} = 2$, as if the 2-edge did not exist—removing them does not affect the interaction type and consequently does not change the value of the likelihood given at Eq. (1). 3-edges can also hide other 3-edges, as depicted in Fig. 2b. Hence, we must bear in mind that we will only be able to make inferences about “visible” hyperedges.

Second, the full model is susceptible to label-switching and thus needs additional adjustments. Indeed, while a non-interacting pair ($\ell _{ij}=0$) and a pair of vertices connected by a 2-edge ($\ell _{ij}=1$) are associated with different distributions of observations because they have distinct means $\mu _0$ and $\mu _1$, it is possible to change the structure *H* and the parameters μ in a way that will not affect the overall likelihood of a dataset *X*. This can be done by replacing every non-interacting pair of *H* by a 2-edge and vice-versa while also swapping the value of $\mu _0$ and $\mu _1$. We address this label-switching symmetry it in a standard way by imposing that $\mu _0<\mu _1$ or, equivalently, by thinking of non-interacting pairs as associated with a smaller expected number of interactions than interacting pairs.

The label $\ell _{ij}=2$ can also technically be exchanged with the labels $\ell _{ij}=0$ and $\ell _{ij}=1$, but because they are inherited from a latent hypergraph that correlates multiple pairs of vertices, the problem will only manifest itself in very specific situations. Namely, every 2-edge has to belong to at least one triangle formed by two other 2-edges or projected 3-edges (this worst-case hypergraph is described in “When are the hyperedges most relevant”). Since a vanishing fraction of hypergraphs exhibit this specific configuration, imposing $\mu _1<\mu _2$ is unnecessary to disambiguate most configurations. That said, in practice, we found it useful to impose $\mu _0<\mu _2$. Type-1 and type-2 interactions are typically sparse, which means that type 0 interactions are dense. Non-interacting pairs could therefore seem to form many triangles and could be interpreted as the projection of 3-edges. Imposing $\mu _0<\mu _2$ avoids any confusion.

**Figure 3:**
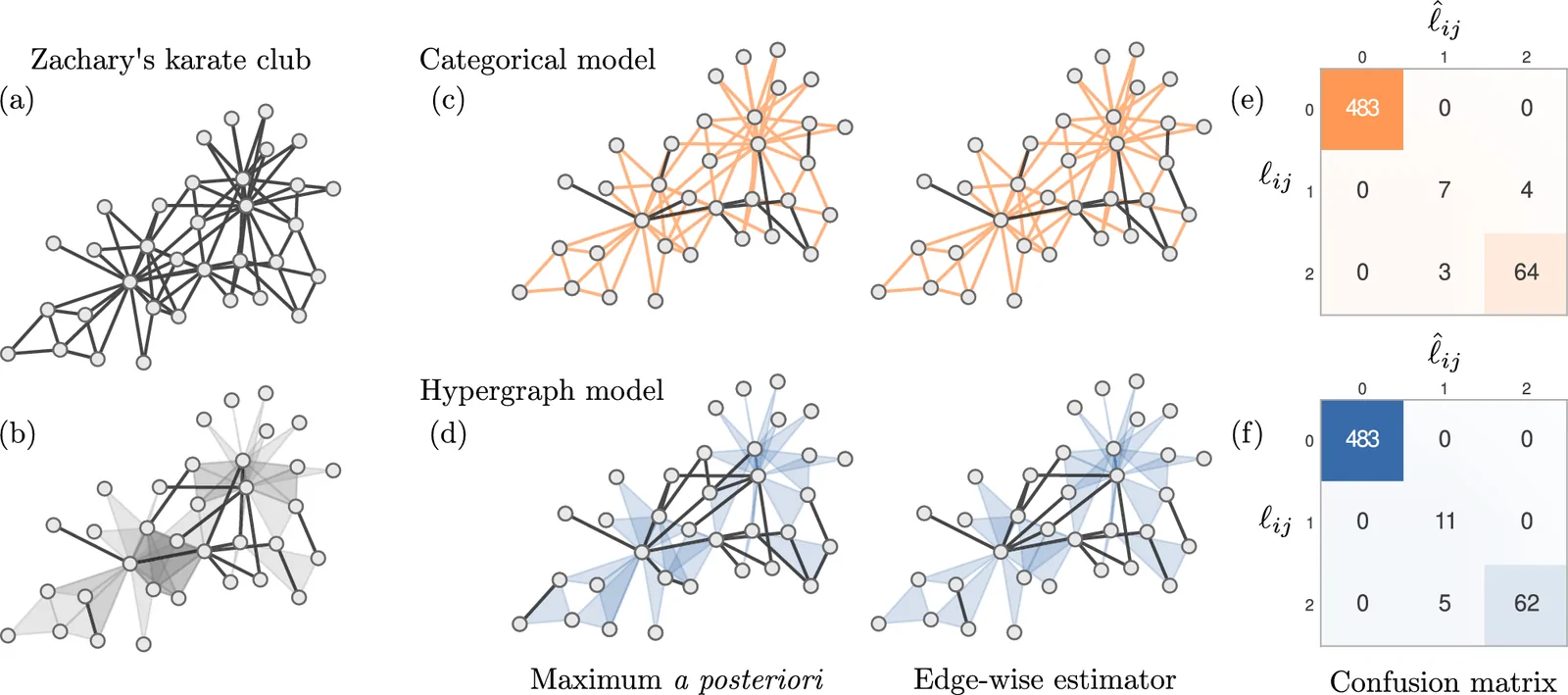
Inference process on a small dataset. (**a**) Original network of Zachary’s karate club^39^. (**b**) Hypergraph representation of the Zachary’s karate club, see main text. (**c**) Illustration of the structure corresponding to the estimators $\hat{\mathcal {S}}_\text {MAP}$ and $\hat{\mathcal {S}}_\text {EW}$ for the categorical-edges model. Strong edges are shown in orange. (**d**) Same as (**c**) but using the hypergraph model. (**e**) Confusion matrix built using the $\hat{S}_\text {MM}$ estimators for the interaction types. (**f**) Same as (**e**) but using the hypergraph model. The inference was done on synthetic observations generated using μ=(0.01,20,30). The maximum *a posteriori* (MAP) structure maximizes the posterior distributions (Eqs. (8) and (9)), while the edge-wise structure contains the edges and hyperedges that exist in at least half of the 500 samples of the posterior distributions. The estimator for the type of interaction, noted $\hat{\ell }_{ij}$, is used to build the confusion matrix. It corresponds to the most likely type of interaction for vertices *i* and *j*.

#### Categorical-edges model

Our second model involves graphs with categorical edges $G=(V, E_1, E_2)$ defined as a set of vertices *V*, of *weak* edges $E_1$, and of *strong* edges $E_2$. The types of interaction are then

5 $$\begin{aligned} \ell _{ij} = {\left\{ \begin{array}{ll} 2 &{} \text {if } (i,j) \in E_2,\\ 1 &{} \text {if } (i,j) \in E_1,\\ 0 &{} \text {otherwise.} \end{array}\right. } \end{aligned}$$

Much like in the hypergraph case, we adopt an agnostic model and assume *a priori* that the categorical edges are placed randomly according to a simple two-step generative process: strong edges are created independently with probability $q_2$ and weak edges are created independently in the remaining unconnected pairs with probability $q_1$

6 $$\begin{aligned} P(G| \phi _G)= & {} q_1^{m_1} (1-q_1)^{\left( {\begin{array}{c}n\\ 2\end{array}}\right) -m_1-m_2} \nonumber \\{} & {} \times q_2^{m_2} (1-q_2)^{\left( {\begin{array}{c}n\\ 2\end{array}}\right) - m_2}, \end{aligned}$$

where $\phi _G=\{q_1,q_2\}$, $m_1=|E_1|$ and $m_2=|E_2|$ are the number of weak edges and strong edges respectively.

There are no hidden edges in this model but the label switching problem is now three-fold: $\ell _{ij}=0$ can be swapped with $\ell _{ij}=1$, but also $\ell _{ij}=0$ with $\ell _{ij}=2$ and $\ell _{ij}=1$ with $\ell _{ij}=2$. Similarly to the hypergraph model, we address this issue by imposing $\mu _0<\mu _1<\mu _2$ since there is no correlation to distinguish $\ell _{ij}=1$ and $\ell _{ij}=2$. Hence, we suppose that non-interacting pairs are less frequently measured than interacting pairs and that weak interactions are less frequently measured than the strong ones.

### Posterior distributions

Combining the quantities defined above, the Bayes formula yields the posterior distribution $P(\mathcal {S}, \mu , \phi |X)$ of each structural model

7 $$\begin{aligned} P(\mathcal {S}, \mu , \phi |X) = \frac{P(X|\mathcal {S},\mu ) P(\mathcal {S}|\phi )P(\mu , \phi )}{P(X)}, \end{aligned}$$

where $P(\mu , \phi )$ is a conjugate prior distribution (see Sect. S1 in Supplementary Material for details) and *P*(*X*) is a normalization factor that needs not to be specified.

Combining Eqs. (2) and (6) with (7) yields the following posterior distributions

8 $$\begin{aligned} P(H, \mu , \phi _H|X)= & {} \frac{P(\mu , \phi )}{P(X)} q^{h_1} (1-q)^{\left( {\begin{array}{c}n\\ 2\end{array}}\right) -h_1} \nonumber \\{} & {} \times p^{h_2} (1-p)^{\left( {\begin{array}{c}n\\ 3\end{array}}\right) -h_2} \prod _{i<j} \frac{ (\mu _{\ell _{ij}})^{x_{ij}} }{x_{ij}!} e^{-\mu _{\ell _{ij}}} \end{aligned}$$

and

9 $$\begin{aligned} P(G, \mu , \phi _G|X)= & {} \frac{P(\mu , \phi )}{P(X)} q_1^{m_1} (1-q_1)^{\left( {\begin{array}{c}n\\ 2\end{array}}\right) -m_1-m_2} \nonumber \\{} & {} \times q_2^{m_2} (1-q_2)^{\left( {\begin{array}{c}n\\ 2\end{array}}\right) - m_2} \prod _{i<j} \frac{ (\mu _{\ell _{ij}})^{x_{ij}} }{x_{ij}!} e^{-\mu _{\ell _{ij}}}, \end{aligned}$$

which both weight every structure-parameters tuple $(S,\mu ,\phi )$ according to their compatibility with the observations *X* and their prior probabilities.

Equations (8) and (9) are not closed forms of known distributions, with the main complication being due to the presence of edge labels $\ell _{ij}$ in the likelihood. Hence, any meaningful use of these posterior distributions will require the generation of samples from it, which in turn will be used to estimate statistics such as percentiles, the average and the variance of various functions $f(\mathcal {S}, \mu , \phi )$. To this end, we have derived Metropolis-within-Gibbs algorithms whose "details are discussed in Sect. S2 in Supplementary Material." The algorithms are initialized from a heuristic for all simulations as described in Sect. S2. A C++/Python implementation is available at https://github.com/DynamicaLab/hypergraph-bayesian-reconstruction. The algorithms return a series of tuples $\{(S_t,\mu _t,\phi _t)\}_{t=1,...N}$ sampled according to Eq. (7) for each structural model.

## Results

### Case study: Zachary’s Karate Club

We first illustrate the framework with a simple case study based on Zachary’s Karate Club^39^. Our goal will be to recover the latent structure of this system, encoded as a hypergraph *H*, given synthetic data *X* generated with the likelihood of Eq. (1) and μ=(0.01,20,30). This μ makes it fairly easy to discern non-interacting pairs but leads to some overlap between the two other types of interactions, which will allow us to highlight the influence of higher-order interactions on the accuracy on the inference (see Fig. 1 which illustrates the distribution of pairwise measurement for this choice of parameters). The structure of the original Karate Club only contains dyadic observations which makes for an uninteresting test of our method, so we add the 3-edges that are found by a separate hypergraph inference technique^32^. (We break down any hyperedge larger than 3 vertices into multiple 3-edges.) We show the original graph and associated hypergraph in Fig. 3a,b—we use the latter throughout our case study.

With this hypergraph structure fixed, we generate a synthetic dataset *X* and approximate its associated posterior distribution using samples generated with the Metropolis-within-Gibbs algorithms. From these samples, we derive two estimators of the structure: the maximum *a posteriori* (MAP) estimator

10 $$\begin{aligned} \hat{\mathcal {S}}_\text {MAP} = \mathop {\textrm{argmax}}\limits \limits _{\mathcal {S}} P(\mathcal {S}|X), \end{aligned}$$

corresponding to the latent structure that maximizes the posterior distribution, and the edge-wise estimator $\hat{\mathcal {S}}_\text {EW}$ that only contains the weak/strong edges or 2-edges/3-edges with a marginal posterior probability above 0.5, e.g., for the hypergraph model

11 $$\begin{aligned} \hat{\mathcal {S}}_\text {EW} = \{ e | \; e \in E\cup T, P(e|X) > 0.5\}, \end{aligned}$$

where *P*(*e*|*X*) is the marginal probability that interaction *e* is present. We complement these structural estimators with an estimator of the type of each pairwise interaction, the maximum marginal estimator

12a $$\begin{aligned} \hat{\mathcal {S}}_\text {MM} = \{\hat{\ell }_{ij}\, | \; i,j \in V\}, \end{aligned}$$

where

12b $$\begin{aligned} \hat{\ell }_{ij} = \mathop {\textrm{argmax}}\limits \limits _{\ell _{ij} \in \{0,1,2\}} P(\ell _{ij}|X) \end{aligned}$$

is the most likely type of interaction type for vertices *i* and *j* (ties are broken by choosing a type at random).

Figure 3c,d show $\hat{\mathcal {S}}_\text {MAP}$ and $\hat{\mathcal {S}}_\text {EW}$ for both models fitted to the same realization of the data *X*. In both cases, we see that our inference framework reconstructs the original structure quite accurately, though both estimators miss a few 3-edges. While some of them are genuine errors, quite a few missing 3-edges are simply hidden and thus unrecoverable (as defined in “Structural models”).

Since these hidden interactions are an artifact of our framework and are therefore irrelevant, we focus on predicting the pairwise interaction types $\ell _{ij}$ which ignore hidden interactions by construction. Figure 3e,f show that both the hypergraph and the categorical-edges model accurately infer the interaction types with the confusion matrix, a generalization of statistical errors (type I and type II errors) for multiple classes. The element $c_{rs}$ of this matrix denotes the number of times a pairwise interaction of type $\ell _{ij}=r$ has been predicted as $\hat{\ell }_{ij}=s$ by the maximum marginal estimator $\hat{\mathcal {S}}_\text {MM}$. Hence, a perfect reconstruction corresponds to a diagonal matrix. The major difference between both confusion matrices is that the categorical-edges model uses weak edges and strong edges somewhat interchangeably, which results in reconstruction errors that go both ways. In contrast, the hypergraph model has no false positive 3-edges. This is due to the restrictive nature of 3-edges: each type-2 pairwise interaction must be associated with at least two other type-2 pairwise interactions (as long as the 3-edge is not hidden). As a result, our framework will err on a more conservative side when assigning larger hyperedges: the framework will assign $\ell _{ij} = 1$ unless there is sufficient evidence in the neighborhood of vertices *i* and *j* that supports a 3-edge. This additional neighborhood information is what allows the hypergraph model to have a smaller sum of off-diagonal elements in the confusion matrix, meaning that it more accurately retrieves the interaction types.

**Figure 4:**
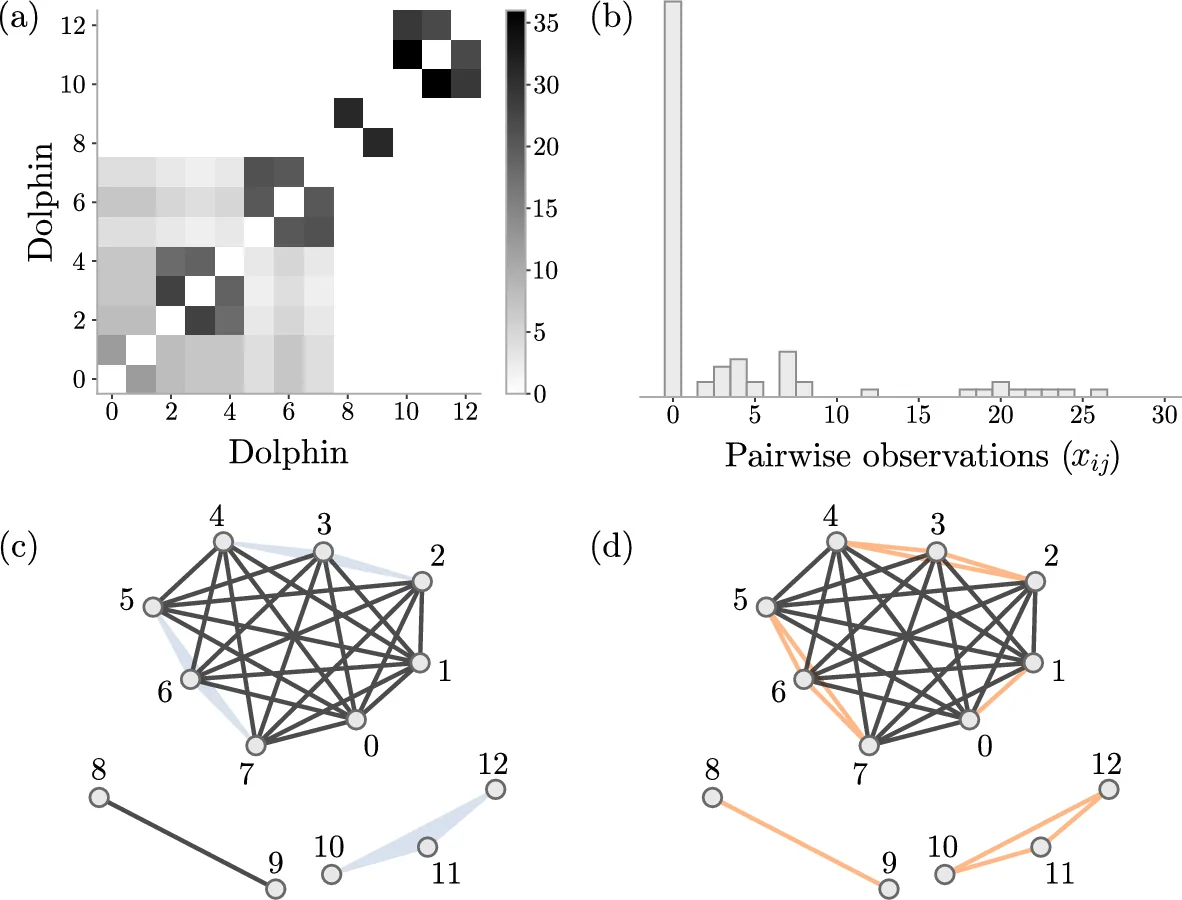
Inference of empirical observations of the interactions of a group of dolphins from Ref.^40^. (**a**) Frequency of each pairwise observation. (**b**) Histogram of the pairwise observations. (**c**) MAP estimator of the hypergraph model. (**d**) MAP estimator of the categorical-edges model. Although observed more frequently, the pair (8, 9) and the pair (0, 1) are inferred as 2-edges (type-1 interaction) in the hypergraph model compared to being classified as type-2 interactions in the categorical-edges model. This is due to the hypergraph model hypothesis which specifies that type-2 interactions must appear as triangles of very frequent pairwise observations (or less frequent if $\mu _2<\mu _1$). The MAP estimators were obtained from a sample of size 500 for each model.

**Figure 5:**
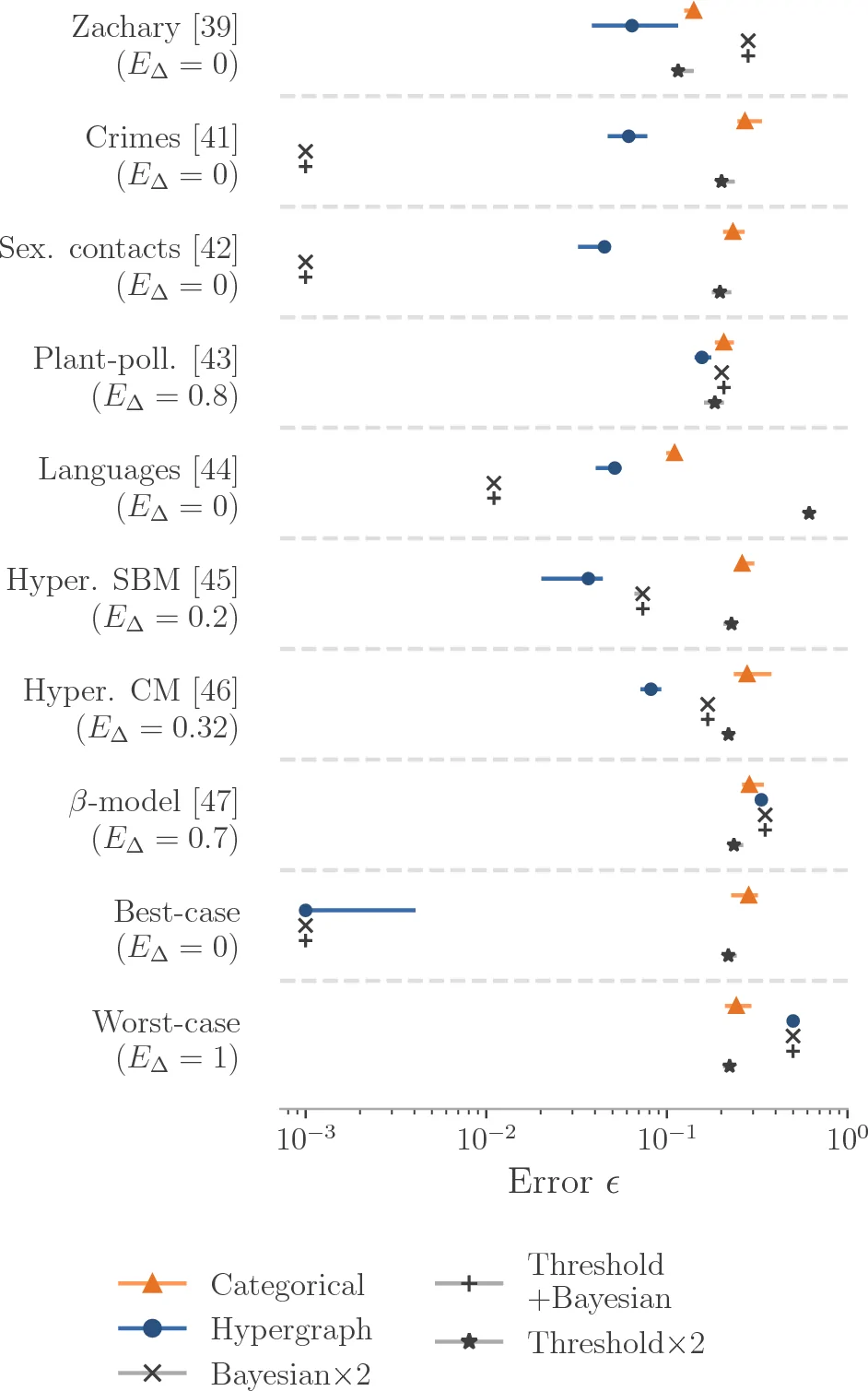
Error of different reconstruction approaches for observations generated both from synthetic and empirical structures. In the simulations, we sample 500 points from the posterior distribution of our Bayesian models, sample 200 points from the posterior distribution of Ref.^19^ and perform 50, 000 swaps of Ref.^32^ algorithm. Points represent the median and errors bars the first and third quartiles for 100 observation matrices generated with μ=(0.01,40,50). Values shown in the figure are bounded below at 0.001 (as the error can be null). The numerical values of this figure are presented in Table S1 of Sect. S3 in Supplementary Material.

### Case study: dolphin interactions

We now apply our framework on observations of 13 male bottlenose dolphins interacting together in a shallow lagoon as they swim^40^.

We first observe that the pairwise observations frequencies shown in Fig. 4b roughly mimic those of the likelihood in Fig. 1, which suggests that the data model introduced in “Data model” is appropriate for this dataset. Looking at Fig. 4a, we also observe that the most frequent pairwise interactions appear in triads of dolphins (i.e. $x_{ij}$, $x_{ik}$ and $x_{jk}$ for dolphins *i*, *j* and *k*) except for dolphins 8 and 9. This suggests that the assumptions behind our hypergraph model are plausible.

Figure 4c,d show that both models predict the same pairwise interactions, but that some inferred types differ. Although dolphins 8 and 9 interacted very frequently, the hypergraph model predicts that they have a type-1 interaction since neither interacted with other dolphins and are therefore unlikely to participate in a 3-edge. In contrast, the categorical-edges model predicts that the pair is a type-2 interaction since it was observed many times. Similarly, the hypergraph model predicts that dolphins 0 and 1 have a type-1 interaction while the categorical-edges model predicts a type-2 interaction. This is because the interactions with other dolphins (i.e., $x_{0k}$ and $x_{1k}$ for k≠0,1) have not been observed frequently enough and, as a result, it is unlikely that a 3-edge connects these dolphins.

For these reasons, the model selection for this dataset is partly guided by the interpretation: if we judge that frequent interactions are mostly explained by triads, then it makes sense to consider pairs of dolphins (0, 1) and (8, 9) as type-1 interactions; otherwise, it makes sense to consider them as type-2 interactions.

Our framework is expected to yield similar results for any empirical dataset whose distribution of pairwise observations is similar to a mixture of Poisson distribution (see Figs. 1 and 4b). Otherwise, the likelihood in Eq. (1) should be adjusted accordingly, as explained previously.

### Comparison to alternative approaches

We compare the performance of our inference framework to other approaches on a broader collection of synthetic and empirical hypergraphs. For the empirical hypergraphs, we select a network of crimes^41^, a network of sexual contacts^42^, a plant–pollinator network^43^ and a network of languages^44^. The original networks are all bipartite, so we again adapt them to our purpose by interpreting one of the two vertex types as hyperedges: individuals are vertices and crimes are hyperedges, sex workers are vertices and hyperedges are their clients, pollinators are vertices and the plants they pollinate are hyperedges, vertices are countries and hyperedges are languages spoken. We ignore hyperedges with more than five vertices to keep a sufficient number of 2-edges in the hypergraph, we project 4-edges and 5-edges to cliques of 3-edges, and we remove any isolated vertex. We also include the hypergraph derived from Zachary’s Karate Club above.

We complement these empirical datasets with hypergraphs generated using the three computer models, namely (i) the superimposed stochastic block model^45^ (two unequal communities of 30 and 70 vertices with connection probabilities of $q_{11}=0.05$, $q_{12}=q_{21}=0.001$ and $q_{22}=0.02$ for 2-edges, and of $p_1=0.005$ and $p_2=0.0001$ for 3-edges inside communities and $p_\text {out}=0.00001$ outside communities), (ii) a triangle-edge configuration model of 100 vertices^46^ (with degrees drawn from independent geometric distributions of means 2 and 3 for 2-edges and 3-edges, respectively), and (iii) the β-model for layered hypergraphs^47^ (with vertex propensities of 2-edges and 3-edges drawn from normal distributions of averages -4.5 and -5 and of standard deviations 2.5 and 2, respectively).

In addition to our Bayesian models, we study 3 alternative reconstruction approaches based on existing methods: 1) place a weak edge if $x_{ij}\ge t_1$ and place a strong edge if $x_{ij}\ge t_2$ (Threshold ×2); 2) place an edge if $x_{ij}\ge t_1$ and infer the hypergraph with Ref.^32^ (Threshold + Bayesian); 3) infer the graph with Ref.^19^ and infer the hypergraph with Ref.^32^ (Bayesian ×2). Here, the hyperedges obtained using Ref.^19^ are projected onto 3-edges to remain consistent with our framework. To maximize the accuracy of these methods and to keep a systematic approach, we set the thresholds $t_1$ and $t_2$ to the theoretical values minimizing the number of misclassified edge types in the limit of large *n*, which is the intersection of two weighted Poisson distributions with weights $\psi _1$ and $\psi _2$ and parameters $\lambda _1$ and $\lambda _2$ respectively (assuming $\lambda _1<\lambda _2$ without loss of generality and $0<\psi _1,\psi _2<1$). We find

13a $$\begin{aligned} t_1&= z\bigg (\mu _0, P(\ell _{ij}=0); \mu _1, P(\ell _{ij}=1)\bigg ) \end{aligned}$$

13b $$\begin{aligned} t_2&= z\bigg (\mu _1, P(\ell _{ij}=1); \mu _2, P(\ell _{ij}=2)\bigg ), \end{aligned}$$

where

14 $$\begin{aligned} z(\lambda _1, \psi _1; \lambda _2, \psi _2) = \frac{\lambda _2-\lambda _1 - \ln \psi _2 + \ln \psi _1}{\ln \lambda _2 - \ln \lambda _1} \end{aligned}$$

is a point located in the interval (e.g., $\big [\lfloor t_1 \rfloor , \lfloor t_1 + 1\rfloor \big ]$) where the two weighted Poisson distributions intersect. The marginal prior probability $P(\ell _{ij}=k)$ for edge-type *k* is set to the proportion of interactions of type *k* in the ground truth hypergraph and the parameters μ are set to the values used to generate the synthetic observations.

As before, we generate a series of synthetic observations with the likelihood in Eq. (1) and μ=(0.01,40,50), and then sample the posterior distribution to compute the confusion matrices of both models. We summarize our results using the fraction of misclassified type-1 and type-2 interactions, a quantity we call the *relative reconstruction error*

15 $$\begin{aligned} \epsilon = \frac{c_{10} + c_{12} + c_{20} + c_{21}}{c_{10} + c_{11} + c_{12} + c_{20} + c_{21} + c_{22}}, \end{aligned}$$

where $c_{rs}$ are the elements of the confusion matrix. This quantity similar to 1-F_1_-score, but it considers both true positives and true negatives (see Appendix S3 A for details).

The results are reported in Fig. 5 where we see that the hypergraph model performs at least as well as the categorical-edges model. We also observe that although the methods based on that of Ref.^32^ sometimes work better, the hypergraph model is never far behind. In fact, we find that overall the hypergraph model shows a good and consistent performance compared to the other methods, thereby making it more reliable. The following section explores the factors influencing the performance of the hypergraph model.

**Figure 6:**
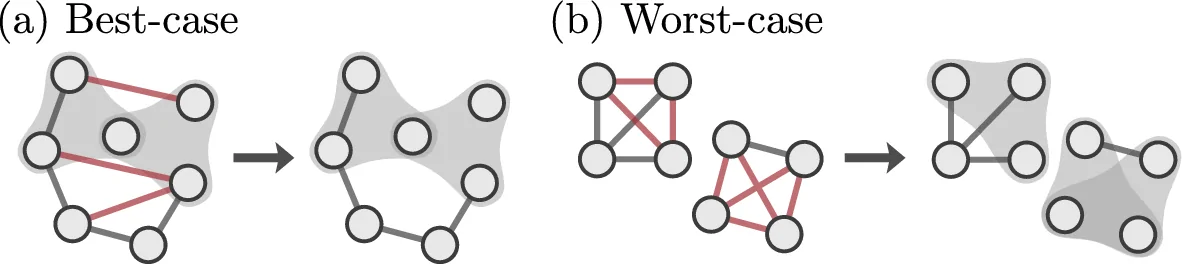
Illustration of the generation of the best-case and worst-case hypergraphs. (**a**) The best-case hypergraphs are obtained by first generating a random hypergraph using Eq. (2) and then removing any 2-edge that creates a triangle when projecting the hypergraph onto the pairwise interactions. While there are many ways to remove 2-edges to respect this constraint, the position of the remaining 2-edges is not important and we therefore only seek to find one solution. (**b**) The worst-case hypergraphs are generated from a graph with isolated cliques of 2-edges and in which each triangle can be promoted to a 3-edge with a given probability.

### When are the hyperedges most relevant

To gain better insights on the factors influencing the performance of the hypergraph model, we consider two extreme cases: a “best-case hypergraph’ and a “worst-case hypergraph”.

In the best-case hypergraphs, groups of 3 vertices can only be connected by a 3-edge. This means that vertices (*i*, *j*, *k*) can form a triangle in projected pairwise interactions only if $\ell _{ij}=\ell _{ik}=\ell _{jk}=2$. As a result, there is no ambiguity on whether or not triangles are a mix of 2-edges and projected 3-edges, and 3-edges can be distinguished from triangles of non-interacting pairs since they have greater pairwise measurements. This effectively makes the neighborhood of any pair of vertices very informative on its type of interaction. We generate such hypergraphs by removing the 2-edges that do not respect the imposed constraint from a hypergraph generated with the prior (2) (see Fig. 6).

The worst-case hypergraphs only contain 2-edges if they form a triangle in the projection. In other words, $\ell _{ij}=1$ is only possible if there exists another vertex *k* such that $\ell _{ik}\ell _{jk}>0$. As a result, the only difference between a type-1 and type-2 interaction (*i*, *j*) is its pairwise observation $x_{ij}$; the neighborhood of a pairwise observation is uninformative. To produce these worst-case hypergraphs, we generate graphs with isolated cliques of 2-edges where each triangle is promoted randomly to a 3-edge (see Fig. 6).

To estimate how much a given hypergraph resembles the best-case or the worst-case, we compute the proportion of 2-edges inside projected triangles

16 $$\begin{aligned} E_\Delta&= \frac{1}{h_1} \sum _{(i,j) \in E} 1 - \prod _{k \in V} 1\!\!1\,[(i,k), (j, k) \not \in \Delta \cup E], \end{aligned}$$

where $1\!\!1$ is the indicator function. The closer $E_\Delta$ is to 0, the closer the hypergraph is to a best-case hypergraph, and the closer the $E_\Delta$ is to 1, the closer the hypergraph is to a worst-case hypergraph.

Revisiting Fig. 5, we see that $E_\Delta$ is related to the error ϵ and that errors for each hypergraph range between the best-case and the worst-case. However, the proportions $\rho _k$ of pairs predicted as type *k*, defined as

17 $$\begin{aligned} \rho _k&= \frac{c_{0k} + c_{1k} + c_{2k}}{\left( {\begin{array}{c}n\\ 2\end{array}}\right) }, \end{aligned}$$

also play a role in ϵ: when a type of interaction is being observed at a similar rate to another, models will most likely favor the type with the largest proportion as it leads to a better fit.

Figure 5 also shows that empirical hypergraphs are generally closer to a best-case hypergraph than to a worst-case. This is due to the sparsity of interactions of empirical complex systems: we expect that most 2-edges are not part of projected 3-edges. For that reason, the hypergraph model works better than the categorical-edges graph model for the majority of systems. And when the hypergraph model errs, both models tend to err as confirmed by the last two lines of Fig. 5.

### Impact of data means

**Figure 7:**
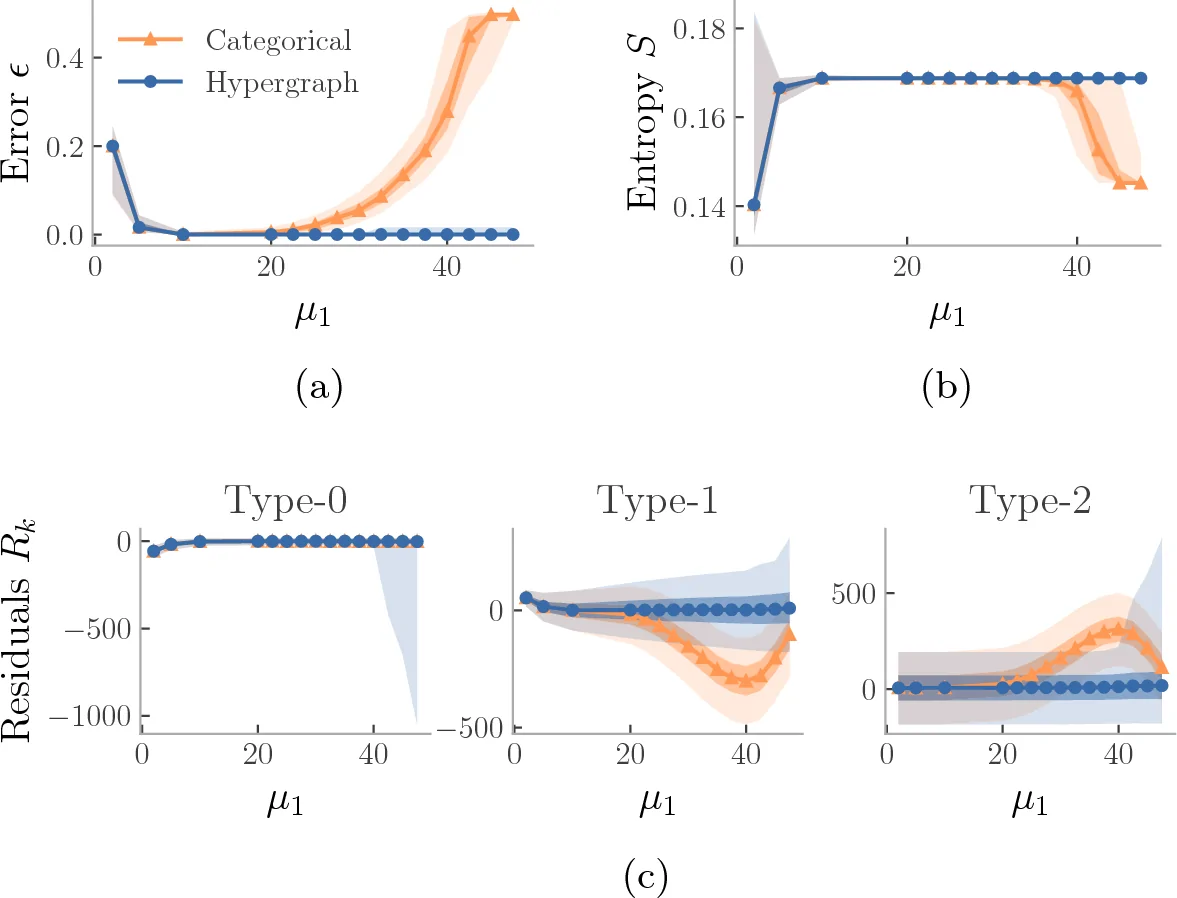
Impact of the measurement rate ($\mu _1$) of type-1 interactions on the reconstruction of a best-case hypergraph. (**a**) Relative reconstruction error ϵ. (**b**) Entropy *S*. (**c**) Sums of residuals $R_k$. The observations were generated with $\mu _0=0.01$, $\mu _2=50$ and various $\mu _1$ using the hypergraph model (blue) and the categorical-edges graph model (orange). The hypergraph model displays (**a**) a smaller reconstruction error (**b**) a larger entropy and (**c**) lower residuals than the categorical-edges graph model, which indicates a better reconstruction. The different statistics are estimated with a sample of size 500. Symbols represent the median, light colored shadings are percentiles 2.5 and 97.5 and dark colored shadings are percentiles 25 and 75 of the metrics for 200 synthetic observations. Residuals were evaluated using 200 predictive observation matrices and the best-case hypergraph was generated using p=0.00017 and q=0.019.

**Figure 8:**
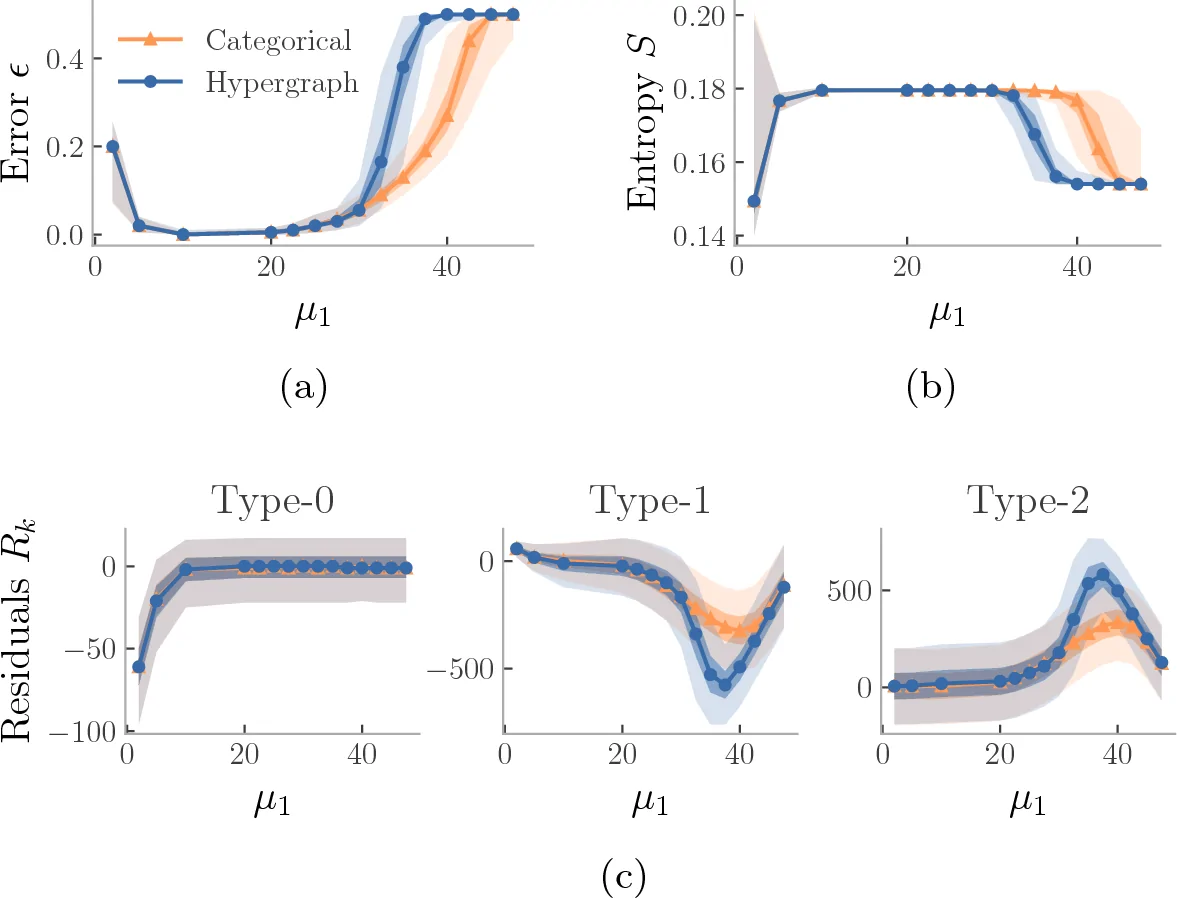
Impact of the measurement rate ($\mu _1$) of type-1 interactions on the reconstruction of a worst-case hypergraph (see Fig. 7 for details). While the categorical-edges graph has a similar performance to the best-case hypergraph (Fig. 7), the hypergraph model cannot distinguish 3-edges from 2-edges with triangles, which results in a worse reconstruction. This is seen with (**a**) a larger reconstruction error (**b**) a smaller entropy and (**c**) larger residuals. The worst-case hypergraph was constructed from 20 isolated 5-cliques in which triangles were promoted to 3-edges with probability 0.19.

To complete our analysis, we study the impact of the parameters μ on the reconstruction by varying $\mu _1$ while keeping $\mu _0=0.01$ and $\mu _2=50$ fixed, for the two families of extreme hypergraphs described above (with n=100 vertices). Doing so allows us to identify the regimes in which the hypergraph model displays a better performance. In addition to the relative reconstruction error ϵ, we also consider two additional summary statistics: the entropy *S* of the label distribution, and the sums of residuals $R_k$.

We define the entropy of the label distribution as

18 $$\begin{aligned} S&= -\sum _{k=0}^2 \rho _k \log _3 \rho _k. \end{aligned}$$

We use $\log _3$ instead of the standard $\log _2$ in information theory for interpretability: the entropy is 0 if only one type of interaction exists and is 1 if the three types are uniformly represented (i.e., $\rho _0=\rho _1=\rho _2=\frac{1}{3}$). Since the empirical datasets are sparse, most pairs of vertices do not interact, meaning that *S* is small. Nevertheless, comparing entropy values allows us to detect when a model completely ignores a type of interaction.

The sums of residuals $R_k$ are defined as

19 $$\begin{aligned} R_k&= \sum _{i<j} (x_{ij} - \tilde{x}_{ij}) \delta _{k,\ell _{ij}}, \end{aligned}$$

where $\tilde{X} = [\tilde{x}_{ij}]_{i,j=1,\dots , n}$ is an observation matrix generated synthetically from the posterior-predictive distribution^19,48^. For each sample point $\mathcal {\tilde{S}}, \tilde{\mu }\sim P(\mathcal {S},\mu |X)$, we generate predictive matrices $\tilde{X}$ from the likelihood (1). This is known as a form of *posterior–predictive check*, and it quantifies the goodness of fit of a model by checking that the fitted model can adequately reproduce the original data. The statistics $R_k$ will reveal biases in the fitted model, with $R_k\approx 0$ only when the predicted pairwise observations $\tilde{x}_{ij}$ are on average equal to the pairwise observations $x_{ij}$ for the interactions of type *k*.

Figures 7 and 8 show that the relative reconstruction error generally increases as $\mu _1$ approaches $\mu _0$ or $\mu _2$. This behavior is expected because there is a greater overlap between the corresponding Poisson distributions in the observations *X*. When this overlap is large, interaction types are represented similarly in the observations *X*, which makes them difficult to infer. Figures 7 and 8 also show that the entropy generally decreases and stabilizes to a lower plateau as $\mu _1$ approaches $\mu _2$. This is due to a similar phenomenon: with the increasing overlap, models favor one type of interaction over the other to the point where one type of interaction disappears. Once the interaction types have “merged”, the entropy remains constant.

For the best-case hypergraph, we clearly see in Fig. 7 that the hypergraph model overall outperforms the categorical-edges graph model. Figure 7a shows that the hypergraph model makes very little reconstruction errors for all sets of parameters. This translates to a higher entropy, as seen in Fig. 7b, and to a smaller predictive bias in Fig. 7c. We conclude that the worse performance observed for the categorical-edges graph model is explained by weak and strong edges ending up being interchangeable because of their pairwise nature. Without the information from the neighborhood that 3-edges imply, the interaction type of a pair $\ell _{ij}$ must be deduced from its observation $x_{ij}$ alone.

For the worst-case hypergraph, Fig. 8 illustrates that the categorical-edges graph model slightly outperforms the hypergraph model. We believe this is due to the prior distribution of the 3-edge probability *p*: because there are $$\left( {\begin{array}{c}n\\ 3\end{array}}\right)$$ possible 3-edges compared to $$\left( {\begin{array}{c}n\\ 2\end{array}}\right)$$ possible 2-edges, there is a much larger number of 3-edges than strong edges for the same probability. In this worst-case setting, 3-edges are almost indistinguishable from 2-edges since triangles are mixture of 2-edges and projected 3-edges. Thus, there is no improvement brought by the hypergraph model, which suggests that this hypergraph representation is not appropriate.

We note that because the Poisson distribution is under-dispersed, the overlap between the edge-type distributions in the data model decreases when the system is observed for a longer time period (i.e. increasing τ when $\mu _1=\tau \lambda _1$ and $\mu _2=\tau \lambda _2$ with $\lambda _1$ and $\lambda _2$ being the measurement rates of the interaction types). As a result, the reconstruction error for both Bayesian models converges to 0 in the limit of large time periods.

## Conclusion

Mounting evidence collected in recent years support that the behavior of many complex systems require taking into account high-order interactions. However, many of the tools of this rapidly expanding field have yet to find practical applications still as measurements of higher-order systems remains challenging to this day.

We presented a minimal Bayesian inference framework that makes progress in this direction, by reconstructing hypergraphs from uncertain observations of their pairwise projection. Using synthetic and empirical datasets, we illustrated the impact that taking into account high-order interactions has on the accuracy of the reconstruction. Notably, we identified the regimes where high-order interactions yield fewer reconstruction errors, due to the fact that hyperedges require the use of local information contained in the neighborhood of vertices.

Although the inference framework introduced here is fairly general, we illustrated it using simple data and hypergraph models to avoid obfuscating its presentation unnecessarily. Thus, future work should be done to apply our framework to hypergraphs with hyperedges larger than 3-edges, and to non-Poissonian data models tailored to other empirical datasets. Doing so will require to treat carefully the way higher-order interactions are assumed to be encoded in the pairwise observation data; as we have shown, insufficient pairwise information may lead to undetectable hyperedges. A partial solution worth investigating involves the use of simplicial complexes, a more restricted higher-order structure in which a hyperedge of size *k* implies every hyperedge of size k-1. Yet, how to connect pairwise interactions to such higher-order interactions remains an open question and is a testament to the bright future Bayesian inference of higher-order interactions has over the coming years.

## Supplementary Information

Supplementary Information.

## Data Availability

All empirical datasets used are available from the cited references.

## Code availability

A C++/Python implementation of our algorithms is available at https://github.com/DynamicaLab/hypergraph-bayesian-reconstruction.
