# Supplementary material for: Hypergraph reconstruction from uncertain pairwise observations
Source: Sci Rep. 2023 Dec 4;13:21364. doi: 10.1038/s41598-023-48081-w (PMC10695935; doi:10.1038/s41598-023-48081-w)
Supplement: Supplementary file 1 — Supplementary Information. [file 41598_2023_48081_MOESM1_ESM.pdf]

Hypergraph reconstruction from uncertain pairwise observations —Supplementary Material—

Simon Lizotte,[1,2] Jean-Gabriel Young,[1,3,4] and Antoine Allard[1,2,4]

[1]*Département de physique, de génie physique et d'optique, Université Laval, Québec (Québec), Canada G1V 0A6*
[2]*Centre interdisciplinaire en modélisation mathématique, Université Laval, Québec (Québec), Canada G1V 0A6*
[3]*Department of Mathematics and Statistics, University of Vermont, Burlington, VT 05405, USA*
[4]*Vermont Complex Systems Center, University of Vermont, Burlington, VT 05405, USA*

S1. PRIOR DISTRIBUTIONS

We use the conjugate priors for each parameter in the model, which correspond to Beta distributions

$$q_1 \sim \mathrm{Beta}(\xi, \zeta) \tag{S1a}$$
$$q_2 \sim \mathrm{Beta}(\xi, \zeta) \tag{S1b}$$
$$p \sim \mathrm{Beta}(\xi, \zeta) \tag{S1c}$$
$$q \sim \mathrm{Beta}(\xi, \zeta). \tag{S1d}$$

In all experiments we set $\xi = 1.1$ and $\zeta = 5$ which encourages sparsity while discouraging the complete removal of a interaction types (a null probability).

As discussed in the main text, we address the potential label switching problem of edge types by imposing an order for the parameters $\mu = (\mu_0, \mu_1, \mu_2)$, which can be viewed as a prior on these parameters [1]. For the categorical-edges model, we impose a total ordering $\mu_0 < \mu_1 < \mu_2$ while the correlations produced by the triangles of the hypergraph model allow us to only assume the partial order $\mu_0 < \mu_1$ and $\mu_0 < \mu_2$ under the assumption that the difference of hyperedge size is sufficient to break symmetries. These considerations translate into the following conjugate distributions for the categorical-edges model

$$\mu_0 \sim \mathrm{Gamma}(\alpha_0, \beta_0) \tag{S2a}$$
$$\mu_1|\mu_0 \sim \mathrm{TruncGamma}_{(\mu_0,\infty)}(\alpha_1, \beta_1) \tag{S2b}$$
$$\mu_2|\mu_1 \sim \mathrm{TruncGamma}_{(\mu_1,\infty)}(\alpha_2, \beta_2), \tag{S2c}$$

and for the hypergraph model we have

$$\mu_0 \sim \mathrm{Gamma}(\alpha_0, \beta_0), \tag{S3a}$$
$$\mu_1|\mu_0 \sim \mathrm{TruncGamma}_{(\mu_0,\infty)}(\alpha_1, \beta_1) \tag{S3b}$$
$$\mu_2|\mu_0 \sim \mathrm{TruncGamma}_{(\mu_0,\infty)}(\alpha_2, \beta_2). \tag{S3c}$$

We use the following probability density functions for $x \sim \mathrm{Gamma}(\alpha, \beta)$ and $y \sim \mathrm{TruncGamma}_{(c,d)}(\alpha, \beta)$:

$$f(x) = \frac{1}{\Gamma(\alpha)} x^{\alpha-1} e^{-\beta x} \tag{S4}$$

$$g(y) = \frac{\mathbb{1}_{(c,d)}(y)}{\gamma(d,\alpha) - \gamma(c,\alpha)} \frac{1}{\Gamma(\alpha)} y^{\alpha-1} e^{-\beta y}, \tag{S5}$$

where γ is the lower incomplete gamma function and $\mathbb{1}$ is the indicator function. Because our synthetic observations are all generated with Poisson distributions with parameter at maximum 50, we set the hyperparameters to $\alpha_0 = 1.0001$, $\alpha_1 = \alpha_2 = 4$, $\beta_0 = 0.5$ and $\beta_1 = \beta_2 = 0.2$ except for the dolphin dataset of section III B where we instead use $\beta_1 = \beta_2 = 0.3$. In another inference setting, these should be adjusted to reflect prior knowledge about the dataset X.

S2. SAMPLING ALGORITHMS

We use a Gibbs sampler to sample the joint posterior distribution $P(\mathcal{S}, \theta|X)$, where $\theta = \{\mu, \phi\}$. This class of algorithms allows us to sample from arbitrary joint distributions by sampling from each of its conditional distributions in alternance, here the parameter distribution $P(\theta|\mathcal{S}, X)$ and the structural distribution $P(\mathcal{S}|X, \theta)$. In what follows, we derive these sampling distributions and determine algorithms that generate samples from them.

A. Sampling the parameters

We break down the sampling of the parameters θ in sequential sampling steps for each of the individual parameters, meaning that when sampling from $P(\theta|\mathcal{S}, X)$, each parameter is conditionally independent to the others. This marginal distribution is noted $P(\theta^*|\theta_{-\theta^*}, \mathcal{S}, X)$ where $\theta_{-\theta^*}$ represents all parameters excluding θ^*. Using Bayes formula, one can see that this distribution is proportional to the posterior distribution

$$P(\theta^*|\theta_{-\theta^*}, \mathcal{S}, X) = \frac{P(\mathcal{S}, \theta|X)}{P(\theta_{-\theta^*}|\mathcal{S}, X) P(\mathcal{S}|X)} \propto P(\mathcal{S}, \theta|X). \tag{S6}$$

Using Eqs. (2), (6) and (S1), we directly find that

$$q_1|\theta_{-q_1}, G, X \sim \mathrm{Beta}(m_1 + \xi, \tbinom{n}{2} - m_1 - m_2 + \zeta) \tag{S7a}$$
$$q_2|\theta_{-q_2}, G, X \sim \mathrm{Beta}(m_2 + \xi, \tbinom{n}{2} - m_2 + \zeta) \tag{S7b}$$
$$q|\theta_{-q}, H, X \sim \mathrm{Beta}(h_1 + \xi, \tbinom{n}{2} - h_1 + \zeta) \tag{S7c}$$
$$p|\theta_{-p}, H, X \sim \mathrm{Beta}(h_2 + \xi, \tbinom{n}{3} - h_2 + \zeta). \tag{S7d}$$

A random variable $z \sim \mathrm{Beta}(a, b)$ can be sampled rapidly with standard univariate sampling methods available in

most statistical software packages, for example as $z = x/(x+y)$ where $x \sim \text{Gamma}(a)$ and $y \sim \text{Gamma}(b)$ [2].

To sample the parameters $\mu = (\mu_0, \mu_1, \mu_2)$, we rearrange the product inside Eq. (1) as

$$P(X|\mathcal{S},\theta) = \prod_{i<j}\left(\frac{1}{x_{ij}!}\right)\prod_{k=0}^{2}\mu_k^{X^{(k)}}e^{-\mu_k L^{(k)}} \tag{S8}$$

where

$$X^{(k)} = \sum_{i<j} x_{ij}\delta_{k,\ell_{ij}} \tag{S9}$$

$$L^{(k)} = \sum_{i<j}\delta_{k,\ell_{ij}} \tag{S10}$$

are, respectively, the sum of observations with edge type k and the number of pairs with edge type k, and where δ is the Kronecker delta. Combining Eqs. (6) and (S6) yields for the categorical-edges graph model

$$\mu_0|\theta_{-\mu_0}, G, X \sim \text{TruncGamma}_{(0,\mu_1)}(X^{(0)}+\alpha_0, L^{(0)}+\beta_0) \tag{S11a}$$

$$\mu_1|\theta_{-\mu_1}, G, X \sim \text{TruncGamma}_{(\mu_0,\mu_2)}(X^{(1)}+\alpha_1, L^{(1)}+\beta_1) \tag{S11b}$$

$$\mu_2|\theta_{-\mu_2}, G, X \sim \text{TruncGamma}_{(\mu_1,\infty)}(X^{(2)}+\alpha_2, L^{(2)}+\beta_2). \tag{S11c}$$

Combining Eqs. (2) and (S6) yields for the hypergraph model

$$\mu_0|\theta_{-\mu_0}, H, X \sim \text{TruncGamma}_{(0,\mu_-)}(X^{(0)}+\alpha_0, L^{(0)}+\beta_0) \tag{S12a}$$

$$\mu_1|\theta_{-\mu_1}, H, X \sim \text{TruncGamma}_{(\mu_0,\infty)}(X^{(1)}+\alpha_1, L^{(1)}+\beta_1) \tag{S12b}$$

$$\mu_2|\theta_{-\mu_2}, H, X \sim \text{TruncGamma}_{(\mu_0,\infty)}(X^{(2)}+\alpha_2, L^{(2)}+\beta_2) \tag{S12c}$$

where $\mu_- = \min\{\mu_1, \mu_2\}$.

Since this step is revisited often by our algorithm, we combine three sampling methods to ensure rapid and accurate sampling in all cases [3]: rejection sampling using a gamma distribution if the rejection probability is low, a more costly inverse transform sampling using incomplete gamma inverse function, and rejection sampling with an adjusted "linear distribution" if all other methods fail. The main interest in using the linear distribution is that it provides a good approximation of the density for small intervals. The inverse transform sampling often works, but can suffer from numerical instabilities especially for small truncation intervals.

We define the linear probability density function as

$$f(x) = \frac{1+cx}{2}, \quad x, c \in [-1,1] \tag{S13}$$

where c is the slope. A sample from this distribution is obtained using its inverse cumulative distribution function

$$\text{CDF}^{-1}(u) = \frac{\sqrt{c^2-2c+4cu+1}-1}{c} \tag{S14}$$

where u is a continuous random variable uniformly distributed on $[0,1]$.

In the rejection sampling algorithm, the support of this distribution is adjusted to match the truncated gamma distribution and c is the slope of a line connecting the truncated gamma density evaluated at the lower bound to the density evaluated at the upper bound.

B. Sampling graphs with categorical edges

The distribution used to sample the categorical-edges graph model is derived by following a similar reasoning as for Eq. (S6). We first observe that

$$P(\mathcal{S}|\theta, X) = \frac{P(\mathcal{S},\theta|X)}{P(\theta|X)} \propto P(\mathcal{S},\theta|X). \tag{S15}$$

Combining this expression with Eqs. (9) and (S1) yields

$$\begin{aligned} P(G|\theta,X) \propto\; & q_1^{m_1+\xi-1}(1-q_1)^{\binom{n}{2}-m_1-m_2+\zeta-1} \\ & \times q_2^{m_2+\xi-1}(1-q_2)^{\binom{n}{2}-m_2+\zeta-1} \\ & \times \prod_{i<j}\frac{(\mu_{\ell_{ij}})^{x_{ij}}}{x_{ij}!}e^{-\mu_{\ell_{ij}}}. \end{aligned} \tag{S16}$$

The edge labels ℓ_{ij} induce complicated interactions between the parameters, so we turn to a Metropolis-Hastings (MH) algorithm to generate samples from this distribution as it does not appear to correspond to a well known closed-form distribution.

The MH algorithm is initialized with a heuristic for all simulations: the initial graph contains weak edges wherever $x_{ij} > 0$ and the initial parameters μ and ϕ_G are set to the maximum likelihood estimator obtained from a Poisson mixture model. At each iteration, we propose to increment a interaction type with probability η and to decrement a interaction type with probability $1-\eta$. We use $\eta = 0.5$ in our numerical simulations.

If the algorithm reaches a point where the graph is fully connected with strong edges (or empty), than we propose to decrement (or increment) a type with probability 1. The pair (i,j) whose type is to be decremented is chosen uniformly among all pairs whose type is not zero. The pair (i,j) whose type is to be incremented is chosen proportionally to the weight

$$w_{ij} = \begin{cases} x_{ij}+1 & \text{if } \ell_{ij} < 2 \\ 0 & \text{otherwise.} \end{cases} \tag{S17}$$

The proposal probability of a new graph G^* conditioned on the current graph G is

$$Q(G^*|G,X) = a\frac{\eta w_{ij}}{\sum_{i<j} w_{ij}} + (1-a)\frac{1-\eta}{m_1+m_2} \tag{S18}$$

where $a = 1$ if the label is to be incremented and $a = 0$ if it is to be decremented. Finally, the proposal is accepted

with probability

$$\alpha(G^*|G) = \min\left(1, \frac{P(G^*,\theta|X)Q(G|G^*,X)}{P(G,\theta|X)Q(G^*|G,X)}\right) \quad \text{(S19)}$$

where $Q(G|G^*,X)$ is the probability of reverting the proposed move.

C. Sampling hypergraphs

Combining Eqs. (8), (S1) and (S15), we find

$$P(H|\theta,X) \propto \frac{P(\theta)}{P(X)} q^{h_1+\xi-1}(1-q)^{\binom{n}{2}-h_1+\zeta-1} p^{h_2+\xi-1} \times (1-p)^{\binom{n}{3}-h_2+\zeta-1} \prod_{i<j} \frac{(\mu_{\ell_{ij}})^{x_{ij}}}{x_{ij}!} e^{-\mu_{\ell_{ij}}}, \quad \text{(S20)}$$

which, again, is not a standard distribution. Hence we use a MH algorithm to generate samples of it in a similar fashion as for the categorical-edges graph model.

The MH algorithm is initialized with a heuristic for all simulations: the initial hypergraph contains 2-edges wherever $x_{ij} > 0$ and the initial parameters μ and ϕ_H are set to the maximum likelihood estimator obtained from a Poisson mixture model. At each iteration, one of six possible moves is proposed:

1. add $(a=1)$ a 2-edge with probability $\nu_2\eta$;
2. remove $(a=0)$ a 2-edge with probability $\nu_2(1-\eta)$;
3. add $(a=1)$ a 3-edge with probability $\nu_3\eta$;
4. remove $(a=0)$ a 3-edge with probability $\nu_3(1-\eta)$;
5. add $(a=1)$ hidden 2-edges with probability $(1-\nu_2-\nu_3)\eta$;
6. remove $(a=0)$ hidden 2-edges with probability $(1-\nu_2-\nu_3)(1-\eta)$.

We use $\eta = 0.5$ and $\nu_2 = \nu_3 = 0.4999$.

If the algorithm reaches a point where either no 2-edge or no 3-edge can be added (removed), then one is removed (added) with probability 1. If a move in which hidden 2-edges should be added/removed has been picked and that move is not possible (e.g. there are no hidden 2-edge to be removed), a completely new move is randomly chosen.

The proposed move, that would transform the hypergraph H into a new one H^*, is accepted with probability

$$\alpha(H^*|H) = \min\left(1, \frac{P(H^*,\theta|X)Q(H|H^*,X)}{P(H,\theta|X)Q(H^*|H,X)}\right). \quad \text{(S21)}$$

We now detail the proposal probability ratio $\frac{Q(H|H^*,X)}{Q(H^*|H,X)}$ for each of the 6 possible moves.

When a 3-edge is to be removed, it is chosen uniformly among the existing 3-edges. When a 3-edge is to be added, the three vertices (i,j,k) are chosen in three steps:

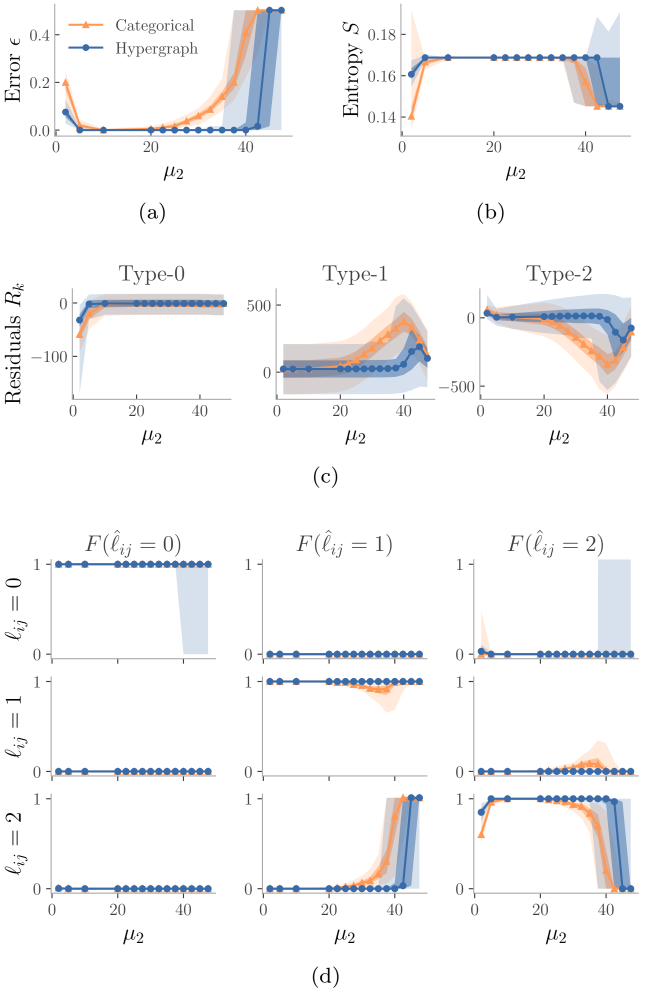

FIG. S1. Impact of the measurement rate (μ_2) of type-2 interactions on the reconstruction of a best-case hypergraph. (a) Relative reconstruction error ϵ. (b) Entropy S. (c) Sums of residuals R_k. (d) Normalized confusion matrix. The observations were generated with $\mu_0 = 0.01$, $\mu_1 = 50$ and various μ_2 using the hypergraph model (blue) and the categorical-edges graph model (orange). The hypergraph model displays (a, d) less reconstruction errors (b) a larger entropy (c) lower residuals than the categorical-edges graph model, which indicates a better reconstruction. See the caption of Fig. 7 for details on the numerical experiment.

pick $i \sim P(i)$, pick $j \sim P(j|i)$ and pick $k \sim P(k|i)$ where

$$P(i) = \frac{\sum_{l\neq i}(x_{il}+1)}{\sum_r \sum_{s\neq r}(x_{rs}+1)} \quad \text{(S22a)}$$

$$P(j|i) = \frac{x_{ij}+1}{\sum_{l\neq i}(x_{il}+1)}. \quad \text{(S22b)}$$

Since the order in which vertices are chosen does not

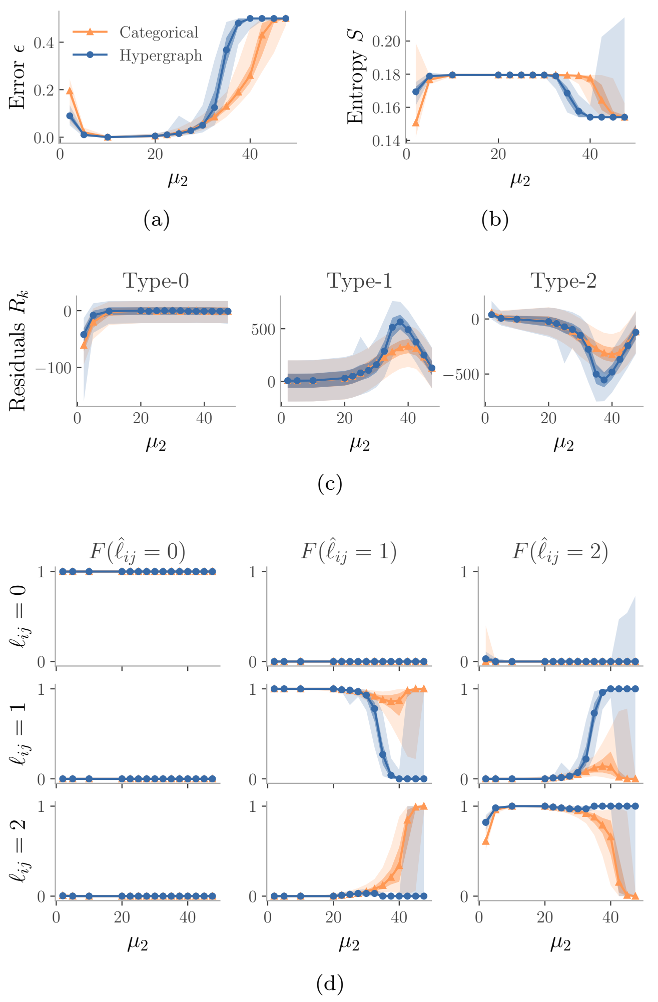

FIG. S2. Impact of the measurement rate (μ_2) of type-2 interactions on the reconstruction of a worst-case hypergraph. (a) Relative reconstruction error ϵ. (b) Entropy S. (c) Sums of residuals R_k. (d) Normalized confusion matrix. The observations are generated with $\mu_0 = 0.01$, $\mu_1 = 50$ and various μ_2 using the hypergraph model (blue) and the categorical-edges graph model (orange). The hypergraph model displays (a, d) more reconstruction errors (b) a smaller entropy (c) greater residuals than the categorical-edges graph model, which indicates a worse reconstruction. See the captions of Figs. 7 and 8 for details on the numerical experiment.

matter, the probability that triplet (i, j, k) is chosen is

$$P(i,j,k) = 2P(i)P(j|i)P(k|i) + 2P(j)P(i|j)P(k|j) + 2P(k)P(i|k)P(j|k). \quad \text{(S23)}$$

If this selection process results in the triplet (i, j, j) or chooses an existing 3-edge, then the proposed move is automatically rejected since the distribution is only supported on *simple* hypergraphs. Altogether, the proposal probability ratio for moves involving 3-edges can be summarized as

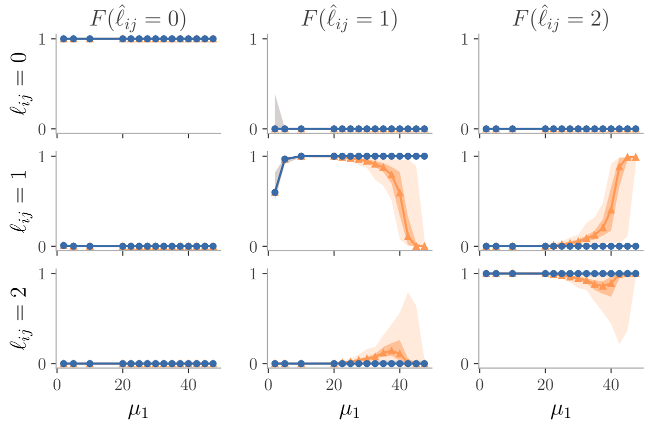

FIG. S3. Normalized confusion matrix associated to the simulation of Fig. 7. The categorical-edges graph model favors the strong edges when μ_1 approaches μ_2, which leads to an inferior reconstruction compared to the hypergraph model that commits little to no error.

$$\frac{Q(H|H^*,X)}{Q(H^*|H,X)} = \left(\frac{1}{\eta P(i,j,k)} \frac{1-\eta}{h_2 + a}\right)^{2a-1}. \quad \text{(S24)}$$

When a 2-edge needs to be removed, it is chosen uniformly among the existing 2-edges. When a 2-edge (i, j) needs to be added, it is chosen proportionally to the weight

$$\omega_{ij} = \begin{cases} x_{ij} + 1 & \text{if } (i,j) \notin E \\ 0 & \text{otherwise.} \end{cases} \quad \text{(S25)}$$

Altogether, the proposal probability ratio for moves involving 2-edges can be summarized as

$$\frac{Q(H|H^*,X)}{Q(H^*|H,X)} = \left(\frac{\sum_{r<s}\omega_{rs} + a(x_{ij}+1)}{\eta(x_{ij}+1)} \frac{1-\eta}{h_1 + a}\right)^{2a-1} \quad \text{(S26)}$$

Our definition of the types of interactions ℓ_{ij} [Eq. (3)] implies that hidden 2-edges do not contribute to the likelihood [Eq. (1)]; their addition/removal depends solely on the hypergraph model. However, the *cost* of removing a 3-edge depends on the number of hidden 2-edges underneath. Because of this asymmetry, we found that running the MH algorithm with the four previous moves tend to get stuck with certain configurations of hidden 2-edges. Our solution has been to propose two additional moves specifically targeting hidden 2-edges.

To propose the addition/removal of hidden 2-edges, we first regroup every existing hidden 2-edges into a set C_0 and every "nonexistent" hidden 2-edges into a set C_1. (These nonexistent hidden 2-edges are interactions of type 2 for which the corresponding 2-edge does not exist.) We then draw the number m of 2-edges to add/remove from a truncated geometric distribution of parameter χ_a

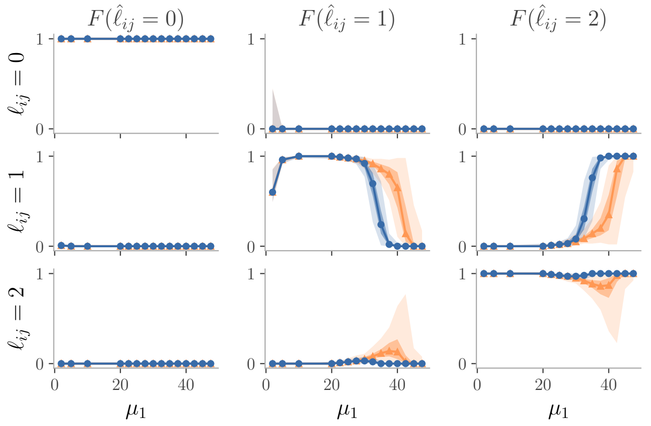

FIG. S4. Normalized confusion matrix associated to the simulation of Fig. 8. While the categorical-edges model still favors strong edges to weak edges, the hypergraph model favors more strongly 3-edges and displays a worse performance for the worst-case hypergraph.

on the interval $[2, |C_a|]$. If $|C_a| < 2$, a new move is picked randomly as the chosen one cannot be performed. We force $m \geq 2$ to ensure these two additional moves do not interfere with the previous two moves involving 2-edges in the MH algorithm acceptance probabilities. Finally, we choose uniformly m hidden 2-edges in C_a and store them in the set e; their addition/removal consist in the proposed move. The probability of a given set e is

$$P(e|C_a, \chi_a) = \frac{(1-\chi_a)^{|e|-2}\chi_a}{1-(1-\chi_a)^{|C_a|-1}} \binom{|C_a|}{|e|}^{-1}, \qquad \text{(S27)}$$

and the proposal probability ratio for moves involving hidden 2-edges only is

$$\frac{Q(H|H^*, X)}{Q(H^*|H, X)} = \left(\frac{1-\eta}{\eta}\right)^{2a-1} \frac{P(e|C_{1-a} \bigcup e, \chi_{1-a})}{P(e|C_a, \chi_a)}. \qquad \text{(S28)}$$

In the simulations, we use $\chi_0 = 0.99$ and $\chi_1 = 0.01$ as we want to remove more frequently than add hidden 2-edges.

D. Convergence and complexity

We stop the two previous MH algorithms whenever the likelihood stabilizes, hinting the chains have reached stationarity. We consider that this has happened when the relative change in the average likelihood of the last W iterations is smaller than a tolerance parameter δ. We use $W = 20000$ and $\delta = 0.02$ in our simulations. When sampling the posterior distribution, we ignore the first iteration given by the Gibbs update as it is slightly affected by our initial condition.

Further, to ensure the MH algorithm runs long enough but not too long, we set a minimum $I_{\min}$ and maximum $I_{\max}$ number of iterations. We adjust these values empirically with a test run, but they are roughly $I_{\min} = 10^5$ and $I_{\max} = 10^6$. Finally, for each posterior distribution sample, we run four chains and keep the one with the highest average likelihood.

Each step in the MH chain runs in linear time with the structure size, and the parameter sampling runs in quadratic time with the structure size. We do not have a firm estimation of the effective time complexity of the structure sampling algorithm because the required number of steps depends on the structure itself.

We would like to stress that, although the quadratic complexity seems expensive, it simply corresponds to number of available pairwise observations. While some authors have proposed to group similar observations to effectively reduce the number of observations, we decided to consider every observation to avoid throwing away information. We would also like to note that the number of steps performed in the MH steps were chosen conservatively; it would be possible to reduce the length of the chains for better performance.

S3. ADDITIONAL RESULTS

A. F_1-scores

The relative reconstruction error defined in the text is similar to the F_1-score, a standard metric corresponding to the harmonic average between the precision and the recall. This quantity can also be expressed as $F_1 = 2TP/(2TP + FP + FN)$ where TP, FP and FN are true positives, false positives and false negatives respectively.

By assigning “true" to correctly predicted edge types, “false" to any incorrect labeling, edge-type 1 to “positives" and edge-type 2 to “negative", the relative reconstruction error of Eq. (15) is equivalent to

$$\epsilon = \frac{FN + FP}{TP + TN + FP + FN} \qquad \text{(S29)}$$

where TN are true negatives. Hence, $1 - F_1 = \epsilon$ if $TP = TN$. Since type-1 (“positives") and type-2 (“negatives") interactions are equally important in our analysis, we used ϵ in the main text. Figure S5 shows that using the F_1 score in our analysis would have yielded the same qualitative observations.

B. Regime $\mu_1 > \mu_2$ and confusion matrices

We mentioned in Sec. II B that conditions such as $\mu_1 < \mu_2$ or $\mu_1 > \mu_2$ need not be imposed in the prior distributions since 2-edges and 3-edges are fundamentally different. As a complement to the analysis presented in Sec. III D, we investigate the case where μ_2 is varied between $\mu_0 = 0.01$ and $\mu_1 = 50$.

Comparison between Figs. 7 and 8 and Figs. S1 and S2 suggests that both parameter orderings are quite similar,

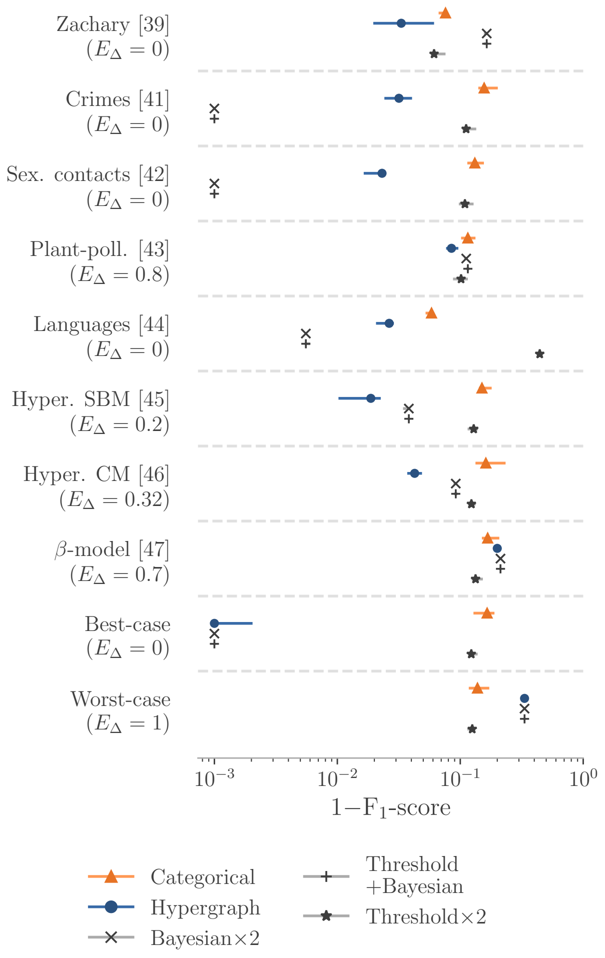

FIG. S5. F_1-scores for the simulations of Fig.5

as expected. In particular, note that the apparent swap in the sums of residuals for the categorical-edges graph model is simply due to the redefinition of ℓ_{ij} to accommodate the restriction that $\mu_1 < \mu_2$ in the model. Indeed we redefine

$$\ell_{ij} = \begin{cases} 1 & (i,j) \in E_2, \\ 2 & (i,j) \in E_1, \\ 0 & \text{otherwise} \end{cases} \tag{S30}$$

for the categorical-edges model.

The only noteworthy difference between the two sets of simulations occurs when μ_2 approaches μ_0. We observe the same phenomenon than when μ_1 approaches μ_2 from the left: the information in the neighborhood used by the hypergraph model allows for a more accurate reconstruction (i.e., smaller ϵ, larger S). Interestingly, this effect is also apparent in the worst-case hypergraph. Indeed, when $\mu_2 \ll \mu_1$, 3-edges can be distinguished from 2-edges with the pairwise observations such that the triangle structure of the worst-case hypergraph is no longer an issue.

Figures S1d, S2d, S3 and S4 show the normalized confusion matrix for the best-case and worst-case hypergraphs. The entries of the normalized confusion matrix $\tilde{c}_{rs}$ are the proportion of interactions of type $\ell_{ij} = r$ that were predicted as $\hat{\ell}_{ij} = s$ by the model

$$\tilde{c}_{rs} = \frac{c_{rs}}{c_{r0} + c_{r1} + c_{r2}}. \tag{S31}$$

For instance, the element $\tilde{c}_{21}$ is the proportion of projected 3-edges predicted as 2-edges in the hypergraph model.

When the categorical-edges graph model ends up inferring only one type of interaction, there are two equivalent reconstructed graphs: all interactions are weak edges or all interactions are strong edges. Noting that in less extreme cases, the model naturally favors strong edges due to the larger associated variance in the likelihood, we set all interactions to strong edges whenever it labels them all as a weak edges.

We see that for the best-case structure in Figs. S1d and S3, the hypergraph model makes little to no error. As we increase μ_1, we also observe a gradual increase of the number of misclassified weak edges for the categorical-edges model. For the worst-case structure, the results in Figs. S2d and S4 show the the hypergraph model favors 3-edges and that the categorical-edges model favors strong edges.

REFERENCES

[1] M. Betancourt, Identifying Bayesian Mixture Models (2017).
[2] J. H. Ahrens and U. Dieter, Computer methods for sampling from gamma, beta, poisson and bionomial distributions, Computing **12**, 223 (1974).
[3] R. J. Gallagher, J.-G. Young, and B. F. Welles, A clarified typology of core-periphery structure in networks, Sci. Adv. **7**, eabc9800 (2021).
[4] J.-G. Young, G. T. Cantwell, and M. E. J. Newman, Bayesian inference of network structure from unreliable data, J. Complex Netw. **8**, cnaa046 (2021).
[5] J.-G. Young, G. Petri, and T. P. Peixoto, Hypergraph reconstruction from network data, Commun. Phys. **4**, 1 (2021).
[6] W. W. Zachary, An Information Flow Model for Conflict and Fission in Small Groups, J. Anthropol. Res. **33**, 452 (1977).
[7] S. H. Decker, C. Kohfeld, R. Rosenfeld, and J. D. Sprague, *The St. Louis Homicide Project: Local Responses to a National Problem* (University of Missouri-St. Louis, 1991).
[8] L. E. C. Rocha, F. Liljeros, and P. Holme, Simulated Epidemics in an Empirical Spatiotemporal Network of 50,185 Sexual Contacts, PLOS Comput. Biol. **7**, e1001109 (2011).
[9] M. Kato, T. Kakutani, T. Inoue, and T. Itino, Insect-flower Relationship in the Primary Beech Forest of Ashu,

TABLE S1. Properties of the synthetic and empirical hypergraph datasets. The table shows the relative reconstruction error (ϵ) for both the categorical-edges graph model and the hypergraph model, when reconstructing edge types with optimal thresholds (13) or an existing Bayesian framework [4] and when using another existing Bayesian framework to infer hyperedges from a graph [5]. The intervals shown here are the first and third quartiles of the relative reconstruction error for 100 observation matrices generated with $\mu = (0.01, 40, 50)$.

	n	type-1	type-2	E_Δ	Categor.	Hyper.	Bayesian ×2	Thres. + Bayes.	Threshold ×2
Zachary [6]	34	11	67	0	0.14(0.12, 0.14)	0.06(0.04, 0.12)	0.28(0.27, 0.28)	0.28(0.27, 0.28)	0.12(0.11, 0.14)
Crimes [7]	202	57	209	0	0.27(0.25, 0.34)	0.06(0.05, 0.08)	0.00(0.00, 0.00)	0.00(0.00, 0.00)	0.20(0.19, 0.24)
Sex. contacts [8]	159	47	108	0	0.23(0.20, 0.27)	0.05(0.03, 0.05)	0.00(0.00, 0.00)	0.00(0.00, 0.00)	0.20(0.18, 0.23)
Plant-poll. [9]	57	51	128	0.80	0.21(0.18, 0.23)	0.16(0.14, 0.18)	0.20(0.19, 0.21)	0.21(0.20, 0.21)	0.18(0.16, 0.21)
Languages [10]	150	30	242	0	0.11(0.10, 0.11)	0.05(0.04, 0.06)	0.01(0.01, 0.01)	0.01(0.01, 0.01)	0.61(0.60, 0.64)
Hyper. SBM [11]	100	60	76	0.20	0.26(0.25, 0.31)	0.04(0.02, 0.04)	0.07(0.07, 0.07)	0.07(0.07, 0.07)	0.23(0.21, 0.24)
Hyper. CM [12]	100	107	89	0.32	0.28(0.23, 0.38)	0.08(0.07, 0.09)	0.17(0.16, 0.17)	0.17(0.16, 0.17)	0.22(0.21, 0.24)
β-model [13]	100	61	56	0.70	0.29(0.26, 0.34)	0.33(0.32, 0.34)	0.35(0.35, 0.36)	0.35(0.35, 0.35)	0.24(0.22, 0.26)
Best-case	100	92	93	0	0.28(0.23, 0.32)	0.00(0.00, 0.00)	0.00(0.00, 0.00)	0.00(0.00, 0.00)	0.22(0.21, 0.24)
Worst-case	100	100	100	1	0.24(0.21, 0.29)	0.50(0.49, 0.50)	0.50(0.50, 0.50)	0.50(0.50, 0.50)	0.22(0.20, 0.23)

Kyoto : An Overview of the Flowering Phenology and the Seasonal Pattern of Insect Visits, Contr. Biol. Lab. Kyoto Univ. **27**, 309 (1990).

[10] J. Kunegis, KONECT: The Koblenz network collection, in *Proceedings of the 22nd International Conference on World Wide Web* (2013) pp. 1343–1350.

[11] S. Paul, O. Milenkovic, and Y. Chen, Higher-Order Spectral Clustering under Superimposed Stochastic Block Model (2018).

[12] J. C. Miller, Percolation and epidemics in random clustered networks, Phys. Rev. E **80**, 020901 (2009).

[13] D. Stasi, K. Sadeghi, A. Rinaldo, S. Petrović, and S. E. Fienberg, β models for random hypergraphs with a given degree sequence (2014).
